# Editorial for Special Issue “Cancer Treatment via Nanotherapy”

**DOI:** 10.3390/nano13071153

**Published:** 2023-03-24

**Authors:** Lisheng Wang

**Affiliations:** 1 Department of Biochemistry, Microbiology and Immunology, Faculty of Medicine, University of Ottawa, 451 Smyth Road, Ottawa, ON K1H 8M5, Canada; lisheng.wang@uottawa.ca; Tel.: +1-613-562-5624; 2 Ottawa Institute of Systems Biology, University of Ottawa, 451 Smyth Road, Ottawa, ON K1H 8M5, Canada; 3 Regenerative Medicine Program, Ottawa Hospital Research Institute, Ottawa, ON K1H 8L6, Canada; 4 Centre for Infection, Immunity and Inflammation, University of Ottawa, 451 Smyth Road, Ottawa, ON K1H 8M5, Canada

Effective cancer treatment remains one of the greatest medical challenges. Nanotherapy, which uses nanomaterials to treat cancers, is an emerging field and a promising alternative to conventional treatments such as surgery, chemotherapy and radiation. In the Special Issue, Cancer Treatment via Nanotherapy, five research papers and four review articles that cover various approaches have been published, reflecting the advances in this field.

Nirachonkul et al. encapsulated anti-CD123 and curcumin into PLGA/poloxamer nanoparticles (CD123-Cur-NPs) to target leukemic stem cells of acute myeloblastic leukemia (AML) [1]. AML exhibits a greater rate of relapse and drug resistance due to the remaining leukemic stem cells after treatment. Leukemic stem cells are quiescent cells and poorly respond to conventional chemotherapeutics, and are capable of self-renewal, resulting in disease relapse. Since CD123 interleukin-3 receptor alpha chain is prominently expressed on CD34^+^CD38^−^ leukemic stem cells but less on normal hematopoietic cells, it was selected in their studies. Curcumin is a bright yellow chemical produced by plants of the *Curcuma longa* species, a member of the ginger family. While curcumin has been reported to inhibit leukemic cell growth, it is unstable, insoluble in water and has low bioavailability. To improve curcumin bioavailability and target leukemia stem cells, Nirachonkul et al. formulated curcumin nanoparticles and conjugated them with CD123 antibody. CD123-Cur-NPs were highly up-taken by KG-1a leukemia cells than Cur-NPs and led to more apoptosis of KG-1a cells. Those nano-drugs, however, did not show cytotoxicity to the normal control hematopoietic cells, providing a promising drug delivery system to improve the therapeutic efficacy against AML.

Asadi et al. evaluated the thermal response of three different NPs (black porous silicon NPs, biodegradable and excretable narrow-NIR-responsive ultrasmall-in-nano architectures, gold nanoparticles) to an 808 nm laser beam with three different powers [2]. The study found that gold nanoparticles have the highest conductivity and diffusivity and that black porous silicon-NPs have the highest radiosensitivity. This study provides important information about the thermal parameters of those NPs for transferring heat to tissue/cancers for pre-clinical planning and applications.

In vitro hyper-harmonized hydroxylated fullerene water complex (3HFWC) has been shown as a promising nanomaterial in cancer treatment. Markelic et al. recently showed that combining 3HFWC with the short-term treatment of cells with hyperpolarized light (HPL) resulted in melanoma cell reprogramming toward a normal phenotype [3]. This suggests that 3HFWC, in combination with HPL, could be used to reprogram tumor cells, providing a potential alternative for cancer treatments. They further determined these effects in vivo in a syngeneic mouse melanoma model [4]. The 3HFWC nanosubstance, in combination with HPL, exhibited in vivo anti-cancer effects. These effects were contributed by the inhibition of melanoma cell growth, the establishment of a senescent phenotype and melanocytic differentiation. In addition, the combined treatment suppressed the pro-tumorigenic activity of immune cells. The 3HFWC nanosubstance, in combination with HPL, inhibited T regulatory cells, myeloid-derived suppressors and M2 macrophages [4]. This could be a new effective approach to inhibit tumor cells while simultaneously improving immune response for cancer treatment.

Chitosan-coated selenium nanoparticles (Cs-SeNPs) have shown potential in cancer treatment, including brain tumors. Dana et al. reported that Cs-SeNPs inhibited cell migration and cell invasion of glioma cells using a trans-well assay. Cs-SeNPs reduced the viability of glioma cells in a dose-dependent manner. Cs-SeNPs also improved the sensitivity of glioma cells to the chemotherapeutic drug 5-FU and had more cellular uptake by glioma cells. Importantly, the Cs-SeNPs labeled with a fluorescent dye were able to pass through the blood–brain barrier in an in vitro model [5]. Those results suggest that Cs-SeNPs could be an effective strategy for future glioma treatment.

Magneto-mechanical therapy has been one of the most promising approaches in tumor microsurgery. Zamay et al. summarized the current progresses in this field [6]. Currently, there are four types of magnetic microdiscs used for tumor destruction: synthetic antiferromagnetic (in-plane), synthetic antiferromagnetic discs (perpendicular), magnetic-vortex microdiscs, and three-layer non-magnetic–ferromagnet–non-magnetic systems with flat quasi-dipole magnetic structures. The most preferred nanodiscs seem to be synthetic antiferromagnetic discs (perpendicular). The authors reviewed the biological effects of those magnetic discs, the mechanisms of action, and the toxicity in alternating or rotating magnetic fields in vitro and in vivo. Current research progress suggests that the targeted and remotely controlled magnetic field nano-scalpel is an effective and safe instrument for cancer therapy or diagnosis.

Among nanoparticles, gold nanoparticles (AuNPs) are one of the most studied and show great promise in cancer therapy [7]. AuNPs have a long circulation time and are easily modified with ligands on cancer cell surface receptors to increase cancer cell uptake through receptor-mediated endocytosis [7]. AuNPs can be used as contrast agents for X-ray-based imaging [7]. AuNP nanocarriers have been synthesized by using nontoxic and biocompatible plants to deliver therapeutic biomolecules for the effective treatment of various cancers. Plant-based synthesized AuNPs provide an approach to developing large-scale production in a greener manner. Fluorescent-plant-based AuNPs have also been used in detecting cancers. Sargazi et al. summarized the benefits of plant-based materials in AuNPs, the application of AuNPs in cancer therapy and detection, and the challenge of AuNPs in drug delivery platforms [7].

Effective drug delivery for cancer therapy is largely affected by the properties of nanoparticles. Substantial achievements in optimizing the structure of nanoparticles for smart drug delivery have been made to date. Jia et al. summarized the optimization strategies of nanoparticles to improve biocompatibility, targeting efficiency, and drug loading rate. This review also provides valuable references for drug delivery of nanoparticles for cancer therapy [8]. 

This Special Issue also included an interesting review article entitled “Modified Bacteriophage for Tumor Detection and Targeted Therapy” [9], which is an emerging area in the cancer research field. Bacteriophages (phages), a natural bacterial virus, have been genetically engineered for use as a probe for the detection of antigens that are highly expressed in tumor cells and as an antitumor reagent. Phages can also be chemically modified and assembled with various nanoparticles to form a new organic or inorganic composite for cancer detection and therapy. Shen et al. summarized the studies on genetically engineered and chemically modified phages in cancer diagnosis and targeted therapy. This review article also discussed the advantages and limitations of modified phages in potential applications. 

Undoubtedly, nanomedicine has become one of the main driving forces in the field to change the current cancer research landscapes, advance cancer treatment, and potentially improve patient outcomes.
